# Technological Strategies to Enhance the Shelf Life of PDO Tuscan Bread in a Compostable Bag-in-Bag System

**DOI:** 10.3390/foods15010065

**Published:** 2025-12-25

**Authors:** Cecilia Akotowaa Offei, Andrea Marianelli, Monica Macaluso, Nicola Mercanti, Bruno Augusto Casu Pereira De Sousa, Marco Alberto Rondanini, Simone Borsani, Angela Zinnai

**Affiliations:** 1 Department of Agriculture, Food and Environment, University of Pisa, Via del Borghetto 80, 56124 Pisa, Italy; ceciliaakotowaa.offei@unito.it (C.A.O.); andrea.marianelli@phd.unipi.it (A.M.); nicola.mercanti@phd.unipi.it (N.M.); angela.zinnai@unipi.it (A.Z.); 2 Department of Agricultural, Forest and Food Sciences, University of Turin, Via Verdi 8, 10124 Torino, Italy; 3 European Laboratory for Nuclear Research (CERN), 1211 Geneva, Switzerland; bruno.casu@cern.ch; 4 INFN Pisa Section, Largo Bruno Pontecorvo 3, 56127 Pisa, Italy; 5 Alithea Srl, Vicolo Gesù 7/9, 20038 Busto Garolfo, Italy; m.rondanini@alithea.it (M.A.R.); s.borsani@alithea.it (S.B.); 6 Interdepartmental Research Centre “Nutraceuticals and Food for Health”, University of Pisa, Via del Borghetto 80, 56124 Pisa, Italy

**Keywords:** food by-product, bakery product, modified atmosphere, shelf life, natural preservatives

## Abstract

Pane Toscano DOP, a traditional sourdough bread from Italy, has a limited shelf life, typically lasting only a few days. Extending its shelf life without the use of synthetic preservatives is essential to meet the rising demand for clean-label products and to reduce food waste. This study aimed to identify the most effective packaging strategy to extend the shelf life of Pane Toscano DOP. Two packaging systems were evaluated: single-package and bag-in-bag systems. In the single-package setup, bread was packaged in PET/PE under different headspace conditions: ambient air (C1), air + Everfresh^®^ Spray (EVF, a natural aromatic extract) (C2), CO_2_ only (C3), and CO_2_ + EVF (C4). In the bag-in-bag system, bread was first placed in a PLA primary package containing air and then enclosed within a PET/PE secondary package filled with either air (T1), air + EVF (T2), CO_2_ only (T3), or CO_2_ + EVF (T4). Shelf life of bread under different packaging conditions was evaluated based on the appearance of visible mold growth. T4 exhibited the longest shelf life, maintaining acceptable quality for 41 days, followed by T3 with 34 days. Air packaged samples C1 and T1 had the shortest shelf life of only 6 days, while C2, T2, and C3 each maintained quality for 20 days. These findings demonstrate that the use of modified atmosphere packaging, bag-in-bag systems, and aromatic headspace extracts can significantly extend the shelf life of artisanal breads, such as Pane Toscano DOP. This approach offers viable alternatives to synthetic preservatives while maintaining traditional product formulations.

## 1. Introduction

Consumption of sourdough-fermented bakery products has grown significantly over the past decade, largely due to their superior health benefits and sensory attributes [1]. Nutritional studies have indicated that sourdough fermentation breaks down proteins, enhancing their digestibility and releasing bioactive peptides and amino acids. Furthermore, sourdough fermentation typically lowers glycemic index, which can be partially attributed to the formation of resistant starches. It also improves the nutritional quality of bread by enhancing the bioavailability of essential micronutrients [2,3]. From a technological point of view, sourdough fermentation improves bread flavor and texture, reduces firmness, and increases loaf volume. These effects are linked to modifications in the gluten network and the production of exopolysaccharides. The distinctive flavor of sourdough bread is primarily derived from volatile compounds such as esters, alcohols, and carbonyls, which are produced during fermentation through amino acid metabolism [2,4].

Italy boasts over 200 types of artisanal or traditional sourdough breads, with several recognized under the EU’s Protected Geographical Indication (PGI) or Protected Designation of Origin (PDO) schemes [1]. One such bread is Pane Toscano, or Tuscan bread, a traditional sourdough product known for its unsalted flavor, hazelnut-like aroma, open crumb structure, crisp crust, and high digestibility. In 2016, Pane Toscano received PDO certification from the European Commission, recognizing the unique qualities linked to its regional origin. The specific climatic conditions of Tuscany influence the properties of the wheat, resulting in flour with ideal hardness, W, and P/L values for Pane Toscano. The bread is prepared with a sourdough starter that ferments unsalted flour over a long period, giving it its unique flavor, texture, and shelf life. Moreover, traditional flour milling techniques that retain the wheat germ also play a critical role in defining its key quality attributes [5].

The shelf life of bread is limited primarily due to fungal spoilage and staling. Sourdough fermentation can offer several benefits by delaying staling through the slowing of starch retrogradation and reduction in crumb firmness [2]. Exopolysaccharides produced during fermentation contribute to this effect by increasing crumb moisture and reducing water loss during storage [4]. In addition, lactic acid bacteria (LAB) generate bacteriocins and antifungal compounds, such as organic acids (acetic, propionic, butyric) and certain dipeptides that help extend shelf life [2].

Nonetheless, artisanal breads like Pane Toscano typically exhibit shorter shelf life than industrial breads, often lasting only a few days. This difference stems from variations in formulation and packaging. Industrial breads frequently contain antifungal agents (e.g., propionates, sorbates), anti-staling enzymes (e.g., lipase, amylase), lipids, and emulsifiers, which collectively prolong shelf life. Artisanal breads, by contrast, are made with fewer ingredients and are generally sold unpackaged or with very simple packaging [6].

Sourdough fermentation provides some natural shelf life extension, but additional strategies are often required to further preserve artisanal breads. One such method is modified atmosphere packaging (MAP), which reduces the oxygen concentration within the package [6]. Since oxygen promotes the growth of aerobic spoilage organisms and facilitates oxidative reactions, lowering its concentration is beneficial [7]. MAP typically employs carbon dioxide, sometimes combined with nitrogen or argon. Carbon dioxide is bacteriostatic and fungistatic, altering the metabolic activity, cell permeability, and intracellular pH of molds [6,7]. MAP has been shown to effectively extend the shelf life of various bread types [8,9,10].

Nevertheless, MAP alone is insufficient to fully prevent spoilage by fungi such as *Endomyces fibuliger* and *Penicillium commune*, which can grow at very low oxygen levels [11,12]. Furthermore, residual oxygen can diffuse from the bread itself or through the packaging over time, creating favorable conditions for microbial growth [13]. Therefore, an additional strategy, such as active packaging, is required to achieve the desired shelf life. In active packaging systems, functional agents are intentionally released into the package to interact with the food or its environment, thereby extending shelf life [6,11]. Effective active packaging methods reported in the literature include oxygen absorbers [13], ethanol emitters [14,15], and systems using natural antimicrobial extracts [11,16].

Beyond active and modified atmosphere packaging strategies, the physical properties of the packaging itself also play a critical role in bread preservation. Barrier properties such as oxygen, carbon dioxide, and water–vapor permeability affect bread staling and microbial growth [17,18]. In this context, multilayer packaging systems with improved barrier properties, through the combination of the strengths of different film layers, have been studied to better preserve food. The overall sensory quality of breads was improved markedly during storage when stored in a multilayer paper package sandwiching starch-NaCl and coated outwardly with a hydrophobic layer compared to a normal paper bag. The modified paper package also reduced the water loss and hardening of the bread during storage [18]. The significance of multilayer packaging in extending the shelf life of bread was further demonstrated by [19]. The packaging system adopting 3-layer LDPE films had a greater effect on extending the microbial shelf life than the use of active packaging (oxygen scavengers) and MAP [19].

Due to the growing demand for clean-label foods and the need to minimize food waste, increasing the shelf life of bakery products without the use of synthetic preservatives has become one of the major concerns of researchers in recent years. Therefore, the objective of this study is to evaluate the most effective packaging strategy for prolonging the shelf life of Pane Toscano DOP. The strategies tested include modified atmosphere packaging using carbon dioxide gas, active packaging, using an aromatic headspace formulation based on natural extracts, and, lastly, a bag-in-bag system.

## 2. Materials and Methods

**Figure 1:**
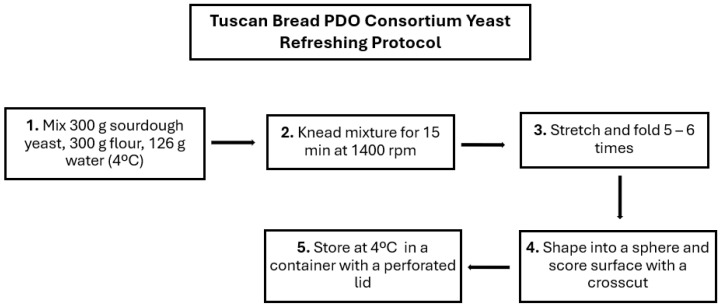
Flow chart of Tuscan Bread PDO Consortium refreshment protocol for type II sourdough yeast detailing the steps.

The Tuscan bread PDO samples (500 g) were produced in triplicate at the production plant of Corsini Bakery Dolci e Biscotti Srl (Castel del Piano, Grosseto, Italy) with Type 0 wheat flour from Molino Angeli Srl (Viareggio, Italy). The sourdough starter culture, used as the leavening agent, was obtained from the Tuscan Bread PDO Consortium (Arezzo, Italy), which was maintained through back-slopping three times a week. The yeast refreshing protocol is represented in Figure 1 below [20]. For the active packaging treatment, the headspace of the bread packages was sprayed with 0.5% m/m Everfresh^®^ Spray (European patent n. 17,195,461 del 18/09/2019 named “Composition for preserving food products” from Alithea Srl, Milan, Italy), a commercial aromatic extract formulation.

It is composed of a mixture of natural volatile compounds predominantly belonging to the classes of monoterpenes, oxygenated monoterpenes, aromatic aldehydes, and phenolic derivatives. These compounds are widely documented for their vapor-phase antifungal activity, acting through disruption of fungal membranes, inhibition of spore germination, and interference with mitochondrial metabolism. Due to the proprietary nature of the formulation, the exact quantitative composition is not publicly disclosed, while the qualitative molecular groups responsible for its antimicrobial effect are described in the patent. The active aromatic fraction typically contains monoterpenes and oxygenated monoterpenes (e.g., citronellal, geraniol, linalool), aromatic aldehydes (e.g., citral, cinnamaldehyde-type–type molecules), and phenolic compounds structurally related to carvacrol/thymol families. Lastly, the packaging materials were furnished by Jolly Plastics S.p.A (Pistoia, Italy).

### 2.1. Storage Experimental Design

The samples were organized based on the packaging test conditions summarized in Table 1. Under each condition, a 500 g loaf of bread was packaged accordingly. All experiments were carried out in four replicates. This resulted in a total of eight experimental groups being shown in Table 1.

This design allowed for a comprehensive evaluation of how the combination of packaging atmosphere, packaging system, and the Everfresh^®^ Spray (EVF) treatment affected the ability to extend the shelf life of the samples during storage. In the bag-in-bag packaging, the inner bag was made of a compostable polylactic acid (PLA) film, while the outer bag consisted of polyethylene terephthalate/polyethylene (PET/PE) films.

The packages were then placed in a temperature-controlled chamber (Model BPC-150F/IC 150-R Plus, Argo Lab, Carpi, Italy) set at 20 °C, with an average ambient humidity of 55%, to minimize temperature-induced humidity fluctuations during subsequent shelf life evaluation. All samples were sealed using a manual heat sealer (Model SALT30, Propac Srl, Rome, Italy).

Table 2 shows the specific characteristics of each packaging material used. Regarding the PET/PE and PLA, we reported the types of structure, coupled layer, type, layer thickness, weight, total weight, water vapor permeability, compostability, and melting point.

To monitor internal conditions throughout storage, a compact, battery-operated sensor device was placed inside each package at the beginning of the experiment until the end of its shelf life. These devices autonomously collected data every 30 min, recording key environmental parameters such as pressure (atm), water vapor content (ppmv), relative humidity (%), and dewpoint (°C). The system was powered by a microcontroller unit (MCU) connected to two digital sensors: the SHT30, MS5803–14BA, similar to a previous paper by [21] but improved to reduce energy consumption and extend battery life, operating in a low-power sleep mode between measurements.

Headspace oxygen and carbon dioxide conditions were monitored daily using the Dansensor CheckPoint^®^ 3 (Model CheckPoint^®^ 3, Ametek Srl, Milan, Italy), and sample weight was also recorded daily until the end of the shelf life period. The shelf life was defined as the number of days from packaging until the first visible fungal growth was observed on the bread surface.

### 2.2. Bread Samples Physicochemical Characterization

The methods to perform physicochemical analyses on these samples were described in a previous study [22], and included

- pH (pH meter, AACC standard methods);
- Total Titratable Acidity (TTA);
- Organic acids (D-/L-lactic, acetic acid) using rapid enzymatic kits (Megazyme Ltd., Bray, Ireland);
- Water activity (Aw), measured using a HygroPalm HP23-AW-A (Rotronic AG, Bassersdorf, Switzerland).

All measurements were carried out in triplicate, ensuring accurate assessment of bread quality, and are shown in Table 3.

### 2.3. Statistical Analysis

All quantitative variables (water vapor content, relative humidity, dew point, oxygen, and carbon dioxide concentrations) were statistically evaluated to determine the effects of packaging condition and storage time. Data was analyzed using a two-way mixed-effects ANOVA model, where packaging condition was treated as a fixed factor and replicate as a random factor to account for intra-treatment variability. When significant main effects or interactions were detected (*p* < 0.05), mean separations were performed using Tukey’s Honest Significant Difference (HSD) post hoc test.

Normality and homogeneity of variance were verified using the Shapiro–Wilk and Levene’s tests, respectively. All results are expressed as mean ± standard deviation (*n* = 4). To ensure data consistency across different degradation phases, data were stratified into two temporal subsets: early storage (0–5 days) and late storage (6–18 days), given that not all packaging treatments reached the end of the experimental period. Statistical computations were performed using GraphPad Prism v. 9.0.0 (GraphPad Software, Boston, MA, USA), and model diagnostics were validated in R (v. 4.3.1; R Core Team, Vienna, Austria).

Shelf life data, defined as the time to visible mold appearance, were treated as right-censored survival data. Kaplan–Meier survival curves were constructed to visualize the probability of samples remaining unspoiled under each packaging condition.

## 3. Results and Discussion

### 3.1. Bread Physicochemical Properties

The physicochemical properties of sourdough breads vary widely based on the formulations and preparation methods used [23]. The measured aw, pH, TTA, lactic acid, and acetic acid of Tuscan bread PDO before packaging (T0) fall within the range commonly observed in wheat-based sourdough breads. Comparable results were reported by [24] in sourdough breads prepared from different starters with aw ranging from 0.90 to 0.81 and acetic acid levels between 0.1234 and 0.0239 g/100 g. However, the lactic acid content was higher than the values obtained in this study, ranging from 0.87224 to 0.4874 g/100 g. Artisanal sourdough bread from central Greece showed a similar pH of 5.7, but it had a higher TTA of 7.9 mL of 0.1N NaOH and aw of 0.981 [25]. Comparable lactic acid levels (0.219 g/100 g) were found in sourdough made through wild microflora fermentation, while higher values (0.259 and 0.296 g/100 g) were reported in breads prepared with lactic acid bacteria inoculum [26]. The presence of organic acids in sourdough bread contributes to its low pH and supports the well-documented extended shelf life that results from the fermentation process.

### 3.2. Evolution of Packaging Internal Environment

The headspace conditions of the packaged breads, including water vapor content (WVC), pressure, relative humidity (RH), and dew point, were monitored until visible mold growth appeared. These measurements were conducted to evaluate how packaging conditions influence the internal environment of the packaged bread and their relationship with shelf life outcomes.

#### 3.2.1. Water Vapor Content

**Figure 2:**
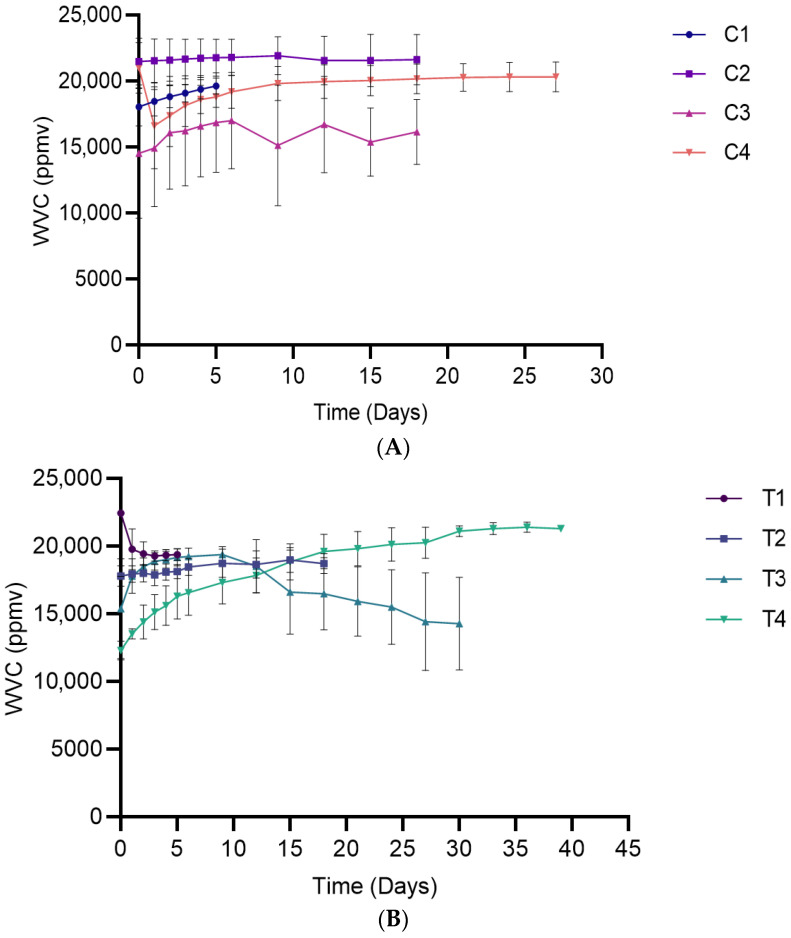
Water vapor content in packaged breads over their respective shelf lives. (**A**) Single packaging system; (**B**) Bag-in-bag packaging system. Data are presented as mean ± SD (*n* = 4). Statistical comparisons (post hoc test results) are presented in Tables S7 and S8 (Supplementary Data).

In the early storage period (0–5 days), storage time had a significant effect on WVC (*p* = 0.0243) (Figure 2). Post hoc results showed significance only in treatments T4 and C1, where higher values were observed on specific days, whereas the remaining packaging types showed no significant variation (Table S7). In the later storage period (6–18 days), storage time had no significant influence on WVC (*p* = 0.8532). This trend may be due to limited moisture migration from the bread matrix into the headspace. Alternatively, it could be related to the water vapor transmission properties of the packaging films, which may have restricted significant changes in WVC during storage.

The packaging condition had a significant effect on the WVC for both the early storage (*p* = 0.0003) and late (*p* = 0.0031) periods studied (Figure 2). Specifically, T4 exhibited significantly lower WVC than the other packaging conditions during the early storage period, while differences among other packaging conditions were less evident (Tables S7 and S8).

#### 3.2.2. Relative Humidity

**Figure 3:**
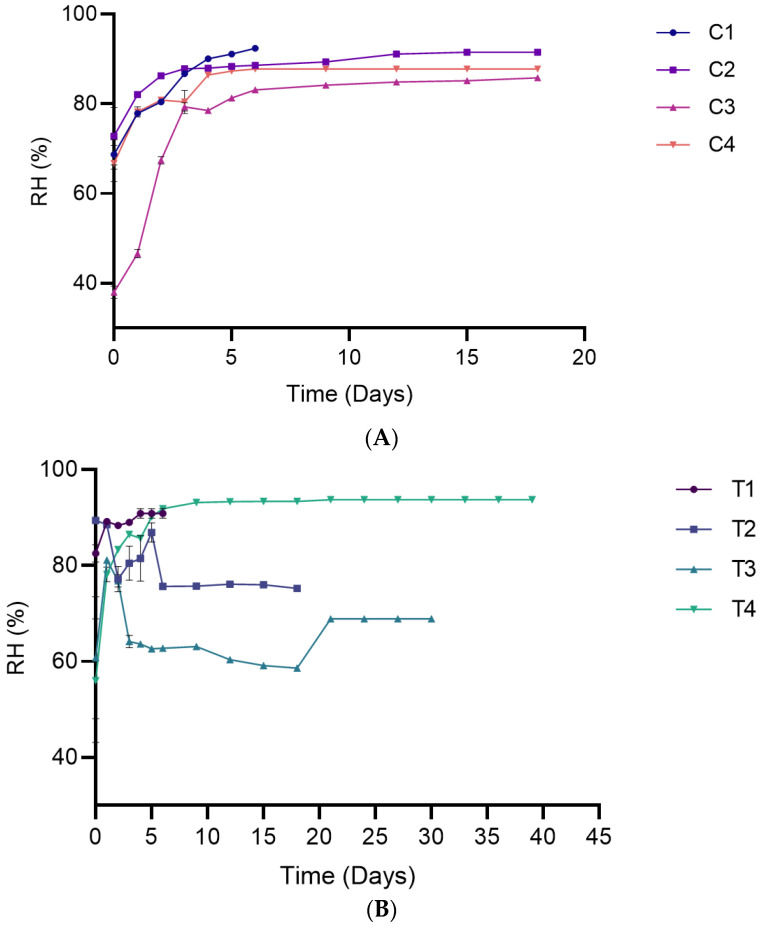
Relative humidity in packaged breads over their respective shelf lives. (**A**) Single packaging system; (**B**) Bag-in-bag packaging system. Data are presented as mean ± SD (*n* = 4). Statistical comparisons (post hoc test results) are presented in Tables S5 and S6 (Supplementary Materials).

Moisture loss from packaged food products during storage elevates the headspace RH [27]. Storage time significantly influenced RH during both the early and late stages of storage (*p* < 0.0001). The RH rose rapidly during the early storage period and then gradually approached a steady state under most packaging conditions (Figure 3). This behavior reflects the high-water activity of the bread (aw = 0.89), which provides a driving force for moisture migration from the bread matrix into the package headspace. Comparable trends have been reported for partly baked Sangak bread, where headspace RH under CO_2_-MAP rapidly increased to a steady state (90–95%), showing the general behavior of headspace RH stabilization over time [28]. In contrast, T2 and T3 showed a sharp decline in RH during the initial storage period, reaching equilibrium later in the shelf life.

Packaging conditions significantly influenced the RH in the bread headspace during the storage periods studied (*p* < 0.0001). Visible mold growth, which defined the end of shelf life in this study, was observed in all samples only after the internal RH had reached a steady state. In the single-packaging system, the effect of the steady state RH value on visible mold onset was evident: lower equilibrium RH values were associated with a longer shelf life, whereas higher steady state RH promoted earlier mold growth. For example, C1, which had the shortest shelf life (6 days), also recorded the highest equilibrium RH (92.370% ± 0.06) than C3 (87.03% ± 0.10) and C4 (87.77% ± 0.00).

Maintaining a lower equilibrium RH is beneficial for product shelf life, as elevated RH combined with minor temperature fluctuations can promote condensation and consequently create favorable conditions for microbial growth [27,29].

The RH values and the other headspace measurements for the bag-in-bag systems primarily represent conditions within the PET/PE headspace, which may not fully reflect the immediate environment surrounding the bread inside the PLA package. This likely explains the differences observed between single and bag-in-bag packaging systems.

#### 3.2.3. Dew Point Temperature

**Figure 4:**
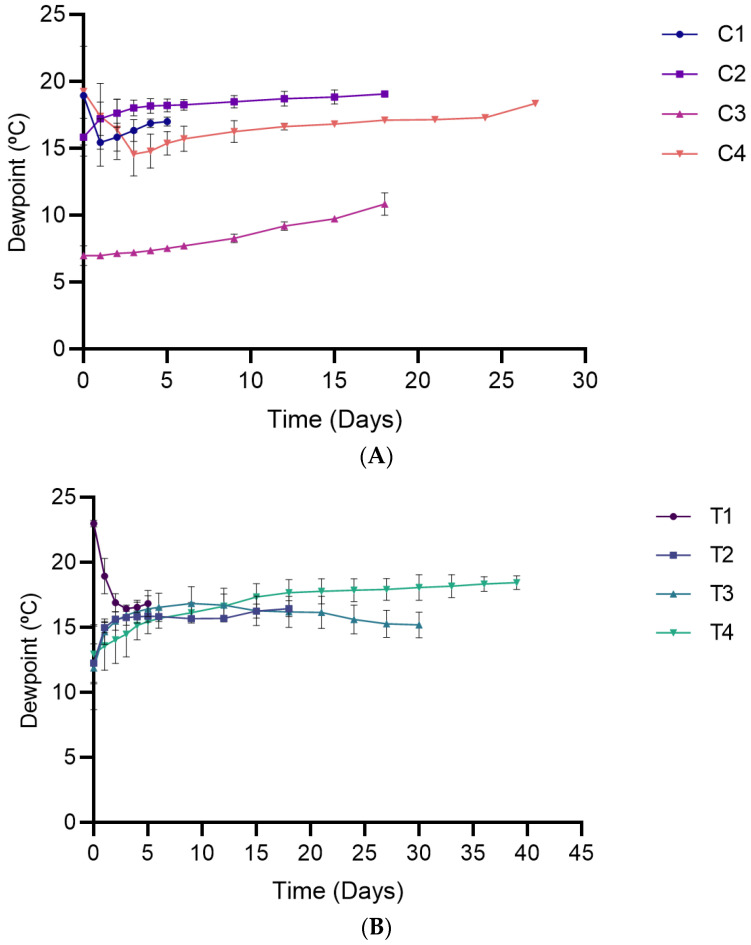
Dew point temperature in packaged breads over their respective shelf lives. (**A**) Single packaging system; (**B**) Bag-in-bag packaging system. Data are presented as mean ± SD (*n* = 4). Statistical comparisons (post hoc test results) are presented in Tables S9 and S10 (Supplementary Materials).

The dew point is the temperature at or below which condensation forms on surfaces [27]. Time did not significantly influence dewpoint temperature (*p* = 0.4435) during the first five days (Figure 4). However, at longer storage durations (6–18 days), a significant effect of time was observed (*p* < 0.0001) where the dew point increased toward 20 °C, approaching the storage temperature. The increase was more evident in C3 than in the other packaging conditions (Table S10). Packaging conditions also significantly influenced the dew point for both storage periods (*p* < 0.0001), with C3 consistently exhibiting significantly lower values among all treatments (Tables S9 and S10).

#### 3.2.4. Pressure

While headspace pressure can be influenced by water vapor, CO_2_, and O_2_ dynamics, it could also serve as an indirect indicator of microbial activity, as gas generation is a common outcome of spoilage [30]. In the present study, the pressure values did not show any significant variations across packaging conditions and time.

### 3.3. Evolution of Headspace Oxygen and Carbon Dioxide Content

**Figure 5:**
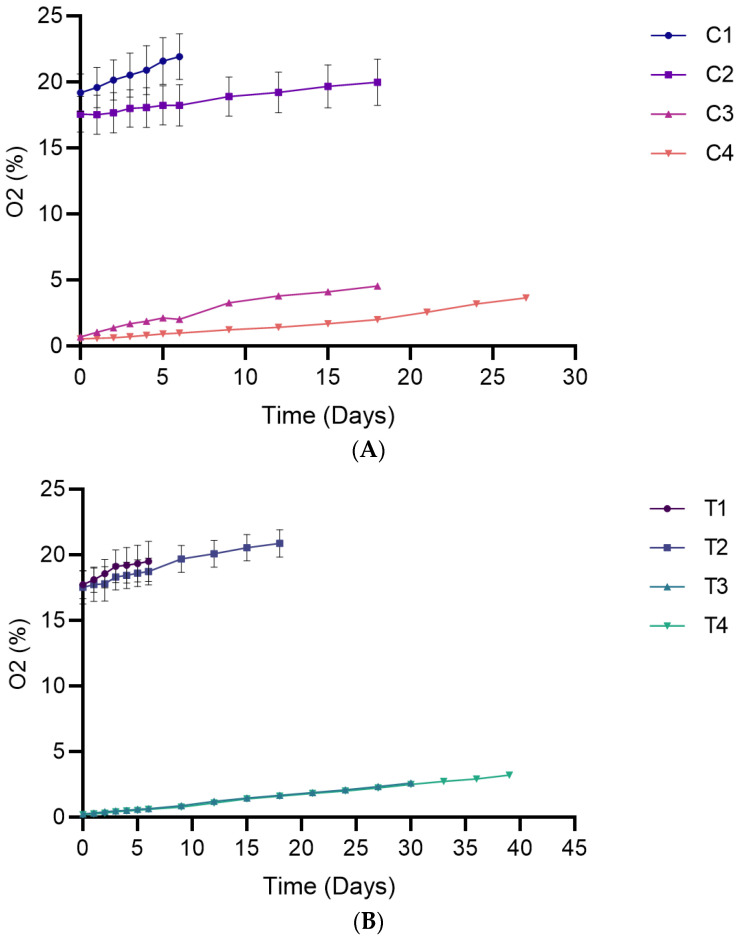
Headspace oxygen levels in packaged breads over their respective shelf lives. (**A**) Single packaging system; (**B**) Bag-in-bag packaging system. Data are presented as mean ± SD (*n* = 4). Statistical comparisons (post hoc test results) are presented in Tables S1 and S2 (Supplementary Materials).

Storage time showed a significant effect on oxygen content in both early (*p* < 0.0001) and late storage periods (*p* < 0.0001). Specifically, the oxygen content increased in packaged breads during storage (Figure 5), although the change was not statistically significant for T1. The observed oxygen increase in packaged breads contrasts with the findings of [31], who reported oxygen depletion in Ciabatta bread under both air and MAP storage conditions, attributing the decline to microbial oxygen consumption. The differences observed may be due to packaging permeability. While their high-barrier films prevented oxygen entry, the films used in this study likely allowed greater oxygen transmission. This allowed for continuous oxygen permeation, which likely obscured the effects of microbial consumption. Additionally, differences in bread type and structure may have also played a role. Supporting this explanation, ref. [19] reported that headspace oxygen in white bread packaged under different oxygen–nitrogen ratios (5:95, 10:90, 21:79) was the net result of oxygen permeation through low-barrier films and microbial consumption. In addition, gas release from internal pores, as reported by [32] in sourdough bread, may also explain the increase in headspace oxygen observed in the present study.

**Figure 6:**
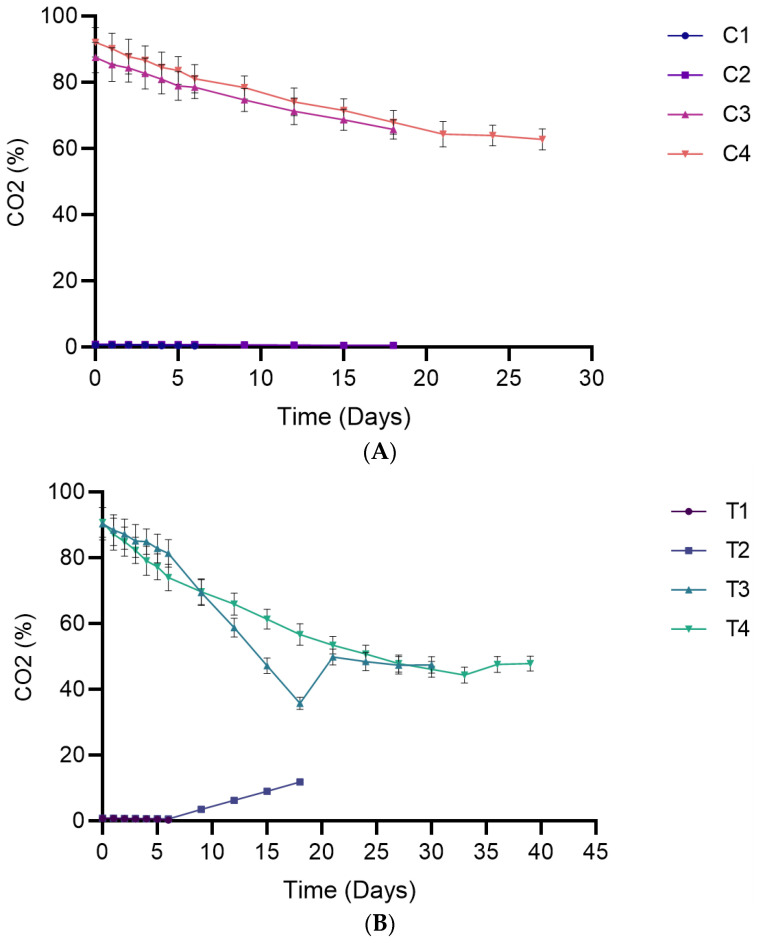
Headspace carbon dioxide levels in packaged breads over their respective shelf lives. (**A**) Single packaging system; (**B**) Bag-in-bag packaging system. Data are presented as mean ± SD (*n* = 4). Statistical comparisons (post hoc test results) are presented in Tables S3 and S4 (Supplementary Materials).

The CO_2_ content of packaged breads gradually declined throughout storage except in T2 (Figure 6). A similar observation was made in traditional Sangak bread by [28]; the researchers attributed the initial decline to the dissolution of the gas in water present in the bread, while the decline at longer storage time was linked to permeation through the packaging material. T2 and T3, showing an increase starting from day 9 and 21, respectively, likely reflect microbial activity, consistent with the findings of [32]. The CO_2_ content of breads stored under CO_2_-MAP (C3, C4, T3, and T4) did not differ significantly up to day 12. After this point, lower CO_2_ levels were observed in the bag-in-bag systems (T3 and T4). These reduced values may reflect the CO_2_ diffusion through the PLA layer into the immediate bread headspace within the inner bag of the bag-in-bag systems (Tables S3 and S4).

### 3.4. Weight Loss

The weight of the packaged breads fluctuated throughout storage, with some increases and decreases observed, and no consistent trend was evident.

### 3.5. Shelf Life

**Figure 7:**
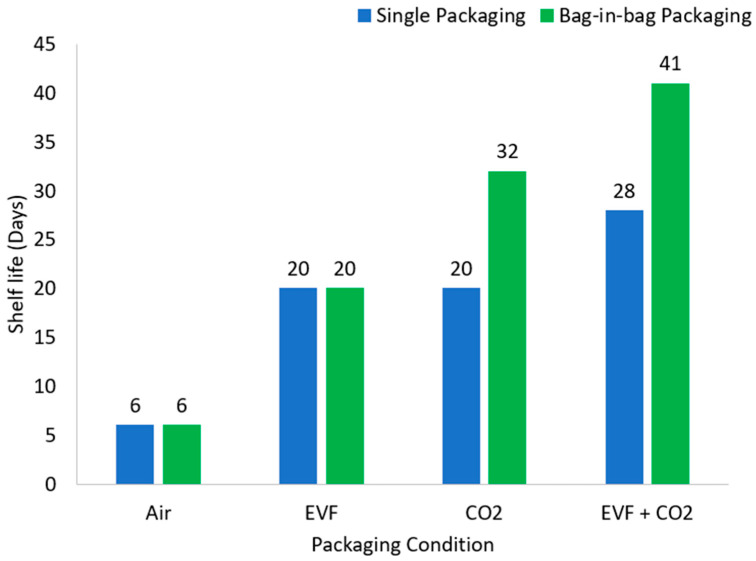
Shelf life of breads packaged under different storage conditions.

The shelf life of the packaged breads was assessed through visual inspection and was defined as the duration until the appearance of visible mold (Figure 7).

Bread samples stored in air without EVF (C1 and T1) exhibited the shortest shelf life, with visible mold growth after six days. This result highlights the crucial role of oxygen in promoting microbial spoilage. Incorporation of EVF in aerobic conditions (C2 and T2) extended shelf life to 20 days. This confirms that vapor-phase release of natural antimicrobials can suppress microbial growth even in the presence of oxygen. These findings align with previous reports where essential oil vapors delayed mold development in bread by 5–15 days [33]; also, in some cases, completely inhibited fungi growth during 14 days of storage [34]. The activity of these extracts is linked to their chemical composition; for example, neral in lemongrass disrupts spore germination, while carvacrol in oregano interferes with cell metabolism [34].

Under MAP with CO_2_ (C3), shelf life improved to 20 days. The inhibitory effect of CO_2_ in MAP is well documented. This effect is attributed to three primary mechanisms: displacement of oxygen in the package headspace, dissolution into the product’s aqueous phase with subsequent formation of carbonic acid, leading to a reduction in pH, and direct interference with microbial membrane function and enzyme systems [19,35]. Similar CO_2_-driven shelf life extensions in bakery products have been documented [7,8,10].

A stronger protective effect was achieved when CO_2_ MAP was combined with EVF (C4), extending shelf life by an additional 8 days. Such synergistic interactions have also been demonstrated in rye, wheat, and gluten-free bakery products where CO_2_ MAP combined with essential oils delayed fungal development more effectively than either strategy alone [36,37].

Importantly, the bag-in-bag system further enhanced mold suppression under CO_2_ (T3) and CO_2_ + EVF (T4) conditions, producing longer shelf lives than their single-bag counterparts (C3 and C4). One likely explanation involves the different gas-permeability properties of the films used in the bag-in-bag system. The PET/PE laminate, with its low CO_2_ permeability, may have retained elevated CO_2_ levels more effectively, whereas the PLA layer, which has a much higher CO_2_ permeability [38], likely allowed gradual CO_2_ diffusion from the outer package into the bread microenvironment. This explanation is consistent with previously reported CO_2_ retention data for the specific films used in this study. PLA films exhibited a loss of 85.76  ±  22.52% of CO_2_ in 6 days, compared with only 8.34  ±  8.38% for PET/PE [21], confirming the substantially greater CO_2_ permeability of PLA films.

It is also plausible that EVF antimicrobial volatiles diffused across the PLA film, as reported by some researchers. Antimicrobial volatiles were detected in the headspace of packaged fresh organic wild rocket [39] and brown rice [40] after diffusing through PLA-based antimicrobial sachets. In the bag-in-bag configuration, this could create a controlled-release effect not present in the single-bag system. Such controlled release and barrier functions allow microbial inhibition without exposing the bread directly to high concentrations of CO_2_ or natural extract vapors, both of which may negatively affect sensory properties. Elevated CO_2_ can increase the perceived acidity of the food [19,41,42], while natural extract residues may compromise consumer acceptability [34,43]. Lastly, the additional PLA layer in the bag-in-bag system likely helped stabilize the microenvironment around the bread by reducing gas exchange with the external environment and buffering against minor temperature or humidity fluctuations.

## 4. Conclusions

The limited shelf life of Pane Toscano DOP highlights the need for effective preservation approaches. This study evaluated CO_2_-based MAP, active packaging with an aromatic headspace formulation from aromatic extracts (Everfresh^®^ Spray), and a bag-in-bag system to determine their effectiveness in extending shelf life.

Pane Toscano DOP stored in air and without EVF had the shortest shelf life, at 6 days, for both bag-in-bag and single packaging systems. The application of EVF alone further extended the shelf life by 14 days for both packaging systems due to its antimicrobial activity. A positive effect of the bag-in-bag system was observed when CO_2_ MAP conditions were applied alone or in combination with EVF. Specifically, compared to their single bag counterparts, the bag-in-bag resulted in shelf life increases of 12 and 13 days in CO_2_ MAP alone and CO_2_ MAP with EVF, respectively. The interpretation that PLA behaves as a more permeable inner layer while PET/PE functions as a high-barrier outer shell is consistent with findings from our previous paper, who compared compostable and conventional bakery packaging films using an MCU-based monitoring system and showed that compostable films such as PLA retained MAP gas compositions less effectively than PET- or PP-based films, indicating higher effective permeability during storage.

This external evidence supports the mechanistic hypothesis proposed here, namely that the differential gas-retention behavior of the two materials contributes to the stabilization of the internal microenvironment in the bag-in-bag configuration.

Accordingly, the extended shelf life observed in T3 and T4 can be interpreted as the result of the complementary barrier properties of the two layers, which together modulate CO_2_ diffusion, humidity dynamics, and active-volatile distribution more effectively than either material alone.

The internal package atmosphere was strongly affected by the complex interaction among bread moisture migration, microbial activity, film permeability (a key limitation of the present study), and the packaging conditions applied. Although the combined application of the bag-in-bag system, EVF, and CO_2_-based MAP showed considerable promise in prolonging bread shelf life, a more detailed understanding of the mechanisms involved will require future studies to use high-barrier films. Such films would reduce confounding permeability effects and allow the dynamics of moisture migration and microbial activity to be more accurately characterized. Incorporating quantitative microbial assessments, texture profiling, and sensory evaluation during storage will also be essential for fully understanding the impact of the applied conditions.

Overall, these findings demonstrate that artisanal bakery products such as Pane Toscano DOP can be successfully preserved using natural packaging strategies, offering a consumer-friendly alternative to conventional chemical preservatives.

## Figures and Tables

**Table 1:** Experimental design plan for storage.

Samples (n_tot_ = 32)	Packaging Conditions
C1	PET/PE + AIR
C2	PET/PE + AIR + 0.5% EVF
C3	PET/PE + 100%CO_2_
C4	PET/PE + 100%CO_2_ + 0.5% EVF
T1	BB + AIR
T2	BB + AIR + 0.5% EVF
T3	BB + 100% CO_2_
T4	BB + 100% CO_2_ + 0.5% EVF

PET/PE: Polyethylene terephthalate/polyethylene; BB: Bag-in-bag packaging system; EVF: Everfresh^®^. For this experiment, Alithea created a specialized Everfresh^®^ Spray with an aromatic head similar to the typical aromatic notes of Tuscan DOP Bread.

**Table 2:** Structural and functional characteristics of PET/PE and PLA-based multilayer packaging films.

Parameter	PET/PE	PLA
Type of structure	Coupled/multilayer	Coupled/multilayer
Outer layer	Polyethylene terephthalate (PET)	Polylactic acid (PLA)
Inner layer	Polyethylene (PE)	Polylactic acid (PLA)
Layer type	Coextruded bioriented	Coextruded bioriented
Thickness of outer layer	20 µm	20 µm
Weight of outer layer	18.2 g/m^2^	18.2 g/m^2^
Thickness of inner (coupled) layer	25 µm	25 µm
Weight of inner layer	22.7 g/m^2^	22.7 g/m^2^
Total weight	45.0 g/m^2^	45.0 g/m^2^
Water vapor permeability (38 °C/90% RH)	5.0 g/m^2^·24 h	5.0 g/m^2^·24 h
Compostability	Non-compostable	Industrially compostable (EN13432)
Melting Point (°C)	250–260	150–170

**Table 3:** Physicochemical parameters of standard Tuscan Bread PDO (T0).

Sample	aw	pH	TTA (mmol/g)	D/L-Lactic Acid (g 100 g^−1^)	Acetic Acid (g 100 g^−1^)
PDO T0	0.89 ± 0.01	5.22 ± 0.03	0.02 ± 0.00	0.28 ± 0.02	0.03 ± 0.01

## Data Availability

The original contributions presented in the study are included in the article/Supplementary Materials, further inquiries can be directed to the corresponding author.

## Supplementary Materials

The following supporting information can be downloaded at https://www.mdpi.com/article/10.3390/foods15010065/s1. Table S1: Percentage oxygen levels in the headspace of packaged breads during the early storage period; Table S2: Percentage oxygen levels in the headspace of packaged breads during the late storage period; Table S3: Percentage carbon dioxide levels in the headspace of packaged breads during the early storage period; Table S4: Percentage carbon dioxide levels in the headspace of packaged breads during the late storage period; Table S5: Percentage relative humidity levels of packaged breads during the early storage period; Table S6: Percentage relative humidity levels of packaged breads during the late storage period; Table S7: Water vapor content in the headspace of packaged breads during the early storage period; Table S8: Water vapor content in the headspace of packaged breads during the late storage period; Table S9: Dew point temperature of packaged breads during the early storage period; Table S10: Dew point temperature of packaged breads during the late storage period.

## Abbreviations

The following abbreviations are used in this manuscript:

MAP	Modified Atmosphere Packaging
PET	Polyethylene terephthalate
PLA	PLA
EVF	Everfresh^®^ Spray
PE	Polyethylene
RH	Relative Humidity
WVC	Water vapor content
