# Supplementary material for: Technological Strategies to Enhance the Shelf Life of PDO Tuscan Bread in a Compostable Bag-in-Bag System
Source: Foods. 2025 Dec 25;15(1):65. doi: 10.3390/foods15010065 (PMC12785392; doi:10.3390/foods15010065)
Supplement: Supplementary file 1 [file foods-15-00065-s001.zip › foods-4023528-supplementary.pdf]

Means, standard deviations, and post-hoc results

Table S1 Percentage oxygen levels in the headspace of packaged breads during the early storage period.

	Packaging Conditions							
**Storage Time**	**C1**	**C2**	**C3**	**C4**	**T1**	**T2**	**T3**	**T4**
**Day 0**	19.198 ± 1.425 ^{dA}	17.568 ± 1.364 ^{deA}	0.708 ± 0.056 ^{dB}	0.543 ± 0.038 ^{eC}	17.742 ± 1.073 ^{aA}	17.522 ± 1.287 ^{defA}	0.263 ± 0.056 ^{gD}	0.2 ± 0.065 ^{dD}
**Day 1**	19.594 ± 1.522 ^{cA}	17.539 ± 1.490 ^{eA}	1.058 ± 0.033 ^{cB}	0.598 ± 0.017 ^{eC}	18.108 ± 0.963 ^{aA}	17.735 ± 1.278 ^{abcA}	0.333 ± 0.054 ^{fD}	0.263 ± 0.056 ^{dD}
**Day 2**	20.160 ± 1.531 ^{bA}	17.679 ± 1.515 ^{cdA}	1.385 ± 0.042 ^{bB}	0.628 ± 0.038 ^{deC}	18.578 ± 1.081 ^{aA}	17.805 ± 1.307 ^{abcA}	0.428 ± 0.065 ^{defD}	0.333 ± 0.054 ^{cD}
**Day 3**	20.537 ± 1.664 ^{bA}	18.003 ± 1.419 ^{abcA}	1.693 ± 0.079 ^{aB}	0.713 ± 0.031 ^{bcdC}	19.129 ± 1.252 ^{aA}	18.313 ± 0.993 ^{cdA}	0.488 ± 0.05 ^{acD}	0.428 ± 0.065 ^{bcD}
**Day 4**	20.897 ± 1.857 ^{bA}	18.074 ± 1.5 ^{bA}	1.888 ± 0.215 ^{abB}	0.813 ± 0.067 ^{acC}	19.229 ± 1.371 ^{aA}	18.455 ± 1.007 ^{beA}	0.523 ± 0.033 ^{bceD}	0.488 ± 0.05 ^{aD}
**Day 5**	21.594 ± 1.771 ^{aA}	18.234 ± 1.479 ^{aA}	2.14 ± 0.227 ^{aB}	0.92 ± 0.028 ^{aC}	19.353 ± 1.413 ^{aA}	18.616 ± 1.02 ^{afA}	0.608 ± 0.031 ^{adD}	0.523 ± 0.033 ^{abD}

For each column, mean values with the same lower-case letters are not statistically significant during storage ($p > 0.05$). For each row, mean values with the same upper-case letters are not statistically significant among the packaging conditions ($p > 0.05$).

Table S2 Percentage oxygen levels in the headspace of packaged breads during the late storage period.

	Packaging Conditions					
**Storage Time**	**C2**	**C3**	**C4**	**T2**	**T3**	**T4**
**Day 6**	18.23 ± 1.573 ^{cA}	2.04 ± 0.474 ^{cB}	0.985 ± 0.017 ^{eB}	18.745 ±1.02 ^{bcdA}	0.665 ± 0.013 ^{eD}	0.608 ± 0.031 ^{eD}
**Day 9**	18.911 ± 1.478 ^{bA}	3.275 ± 0.07 ^{cB}	1.233 ± 0.026 ^{dC}	19.708 ±1.039 ^{cA}	0.885 ± 0.013 ^{dD}	0.785 ± 0.024 ^{dE}
**Day 12**	19.22 ±1.55 ^{bA}	3.808 ± 0.09 ^{bB}	1.425 ± 0.025 ^{cC}	20.096 ±1.031 ^{bA}	1.225 ± 0.034 ^{cD}	1.095 ± 0.021 ^{cE}
**Day 15**	19.68 ±1.625 ^{aA}	4.12 ± 0.037 ^{bB}	1.708 ± 0.025 ^{bC}	20.544 ±1.018 ^{adA}	1.473 ± 0.066 ^{bD}	1.398 ± 0.057 ^{bD}
**Day 18**	19.987 ± 1.743 ^{aA}	4.55 ± 0.026 ^{aB}	2.003 ± 0.03 ^{aC}	20.891 ±1.053 ^{aA}	1.675 ± 0.037 ^{aD}	1.618 ± 0.029 ^{aD}

For each column, mean values with the same lower-case letters are not statistically significant during storage ($p > 0.05$). For each row, mean values with the same upper-case letters are not statistically significant among the packaging conditions ($p > 0.05$).

Table S3 Percentage carbon dioxide levels in the headspace of packaged breads during the early storage period.

	Packaging Conditions							
**Storage Time**	**C1**	**C2**	**C3**	**C4**	**T1**	**T2**	**T3**	**T4**
**Day 0**	0.666 ± 0.032 ^{aA}	0.855 ± 0.044 ^{aB}	87.546 ± 4.615 ^{aC}	92.11 ± 4.429 ^{aC}	0.828 ± 0.047 ^{aB}	0.899 ± 0.05 ^{aB}	90.349 ± 4.977 ^{aC}	90.765 ± 4.489 ^{aC}
**Day 1**	0.657 ± 0.037 ^{aA}	0.839 ± 0.044 ^{bB}	85.395 ± 5.068 ^{bC}	90.173 ± 4.741 ^{bC}	0.78 ± 0.04 ^{bB}	0.871 ±0.04 ^{aB}	88.492 ± 4.696 ^{aC}	87.248 ± 4.913 ^{bC}
**Day 2**	0.637 ± 0.038 ^{bA}	0.824 ± 0.037 ^{bB}	84.357 ± 4.246 ^{bcC}	87.79 ± 5.244 ^{cC}	0.718 ± 0.038 ^{cAB}	0.826 ± 0.043 ^{bB}	87.203 ± 4.627 ^{bC}	84.984 ± 4.41 ^{cC}
**Day 3**	0.640 ± 0.033 ^{bA}	0.806 ± 0.038 ^{cB}	82.698 ± 4.674 ^{cC}	86.75 ± 4.404 ^{cC}	0.654 ± 0.04 ^{dAD}	0.777 ± 0.046 ^{cBD}	85.162 ± 5.03 ^{cC}	82.3 ± 4.005 ^{dC}
**Day 4**	0.499 ± 0.027 ^{cA}	0.781 ±0.04 ^{dB}	80.987 ± 4.453 ^{dC}	84.546 ± 4.598 ^{dC}	0.602 ± 0.035 ^{eD}	0.749 ± 0.037 ^{dB}	84.920 ± 3.896 ^{cC}	79.155 ± 4.451 ^{eC}
**Day 5**	0.475 ± 0.03 ^{dA}	0.755 ± 0.046 ^{eB}	79.00 ± 4.361 ^{eC}	83.629 ± 4.138 ^{dC}	0.549 ± 0.026 ^{fA}	0.704 ± 0.038 ^{eB}	82.855 ± 4.377 ^{dC}	77.259 ± 3.917 ^{eC}

For each column, mean values with the same lower-case letters are not statistically significant during storage ($p > 0.05$). For each row, mean values with the same upper-case letters are not statistically significant among the packaging conditions ($p > 0.05$).

Table S4 Percentage carbon dioxide levels in the headspace of packaged breads during the late storage period.

	Packaging Conditions					
**Storage Time**	**C2**	**C3**	**C4**	**T2**	**T3**	**T4**
**Day 6**	0.752 ± 0.035 ^{aA}	78.55 ± 3.419 ^{aB}	81.102 ± 4.247 ^{aB}	0.662 ± 0.039 ^{aA}	81.396 ± 4.2 ^{aB}	74.081 ± 3.998 ^{aB}
**Day 9**	0.689 ± 0.034 ^{bA}	74.747 ± 3.578 ^{bB}	78.42 ± 3.549 ^{bB}	3.504 ± 0.169 ^{bC}	69.451 ± 3.905 ^{bB}	69.747 ± 3.952 ^{bB}
**Day 12**	0.632 ± 0.034 ^{cA}	71.282 ± 4.035 ^{cB}	74.111 ± 4.178 ^{cB}	6.302 ± 0.344 ^{cC}	58.815 ± 2.843 ^{cD}	65.978 ± 3.342 ^{cBD}
**Day 15**	0.58 ± 0.031 ^{dA}	68.738 ± 3.188 ^{dBE}	71.502 ± 3.499 ^{dB}	9.052 ± 0.548 ^{dC}	47.168 ± 2.39 ^{dD}	61.387 ± 3.052 ^{dE}
**Day 18**	0.524 ± 0.028 ^{eA}	65.785 ± 2.908 ^{eB}	67.958 ± 3.558 ^{eB}	11.864 ± 0.731 ^{eC}	35.794 ± 1.803 ^{eD}	56.726 ± 3.279 ^{eE}

For each column, mean values with the same lower-case letters are not statistically significant during storage ($p > 0.05$). For each row, mean values with the same upper-case letters are not statistically significant among the packaging conditions ($p > 0.05$).

Table S5 Percentage relative humidity levels of packaged breads during the early storage period.

	Packaging Conditions							
**Storage Time**	**C1**	**C2**	**C3**	**C4**	**T1**	**T2**	**T3**	**T4**
**Day 0**	68.723 ± 3.313 ^{eA}	72.808 ± 6.418 ^{abcdACD}	37.948 ± 1.338 ^{fB}	66.743 ± 4.068 ^{dA}	82.538 ± 1.788 ^{cC}	89.438 ± 0.38 ^{abD}	60.753 ± 12.713 ^{abcABCD}	56.005 ±12.904 ^{abcdABCD}
**Day 1**	77.88 ± 0.527 ^{dA}	82.09 ± 0.414 ^{dB}	46.598 ± 0.944 ^{eC}	78.19 ± 1.118 ^{dAD}	89.168 ± 0.432 ^{abE}	88.535 ± 0.051 ^{abE}	81.153 ± 0.658 ^{aBD}	78.145 ±1.532 ^{dAB}
**Day 2**	80.413 ± 0.158 ^{cAE}	86.278 ± 0.138 ^{cB}	67.405± 0.805 ^{dC}	80.855 ± 0.237 ^{cA}	88.368 ± 0.197 ^{bD}	77.145 ± 2.632 ^{cdAEF}	76.788 ± 1.337 ^{aE}	83.368 ± 0.448 ^{cF}
**Day 3**	86.728 ± 0.190 ^{bA}	87.86 ± 0.191 ^{bB}	79.34 ± 0.955 ^{bcC}	80.44 ± 2.568 ^{abcdABC}	88.983 ± 0.106 ^{aD}	80.478 ± 3.55 ^{bcABCD}	64.18 ± 1.323 ^{bcE}	86.52 ± 0.296 ^{bA}
**Day 4**	90.03 ± 0.184 ^{aA}	87.935 ± 0.031 ^{bB}	78.485 ± 0.389 ^{bC}	86.433 ± 0.1 ^{bD}	90.888 ± 1.006 ^{aAB}	81.483 ± 4.752 ^{abdABCD}	63.63 ± 0.168 ^{bE}	85.678 ± 0.632 ^{bD}
**Day 5**	91.105 ± 0.319 ^{aA}	88.368 ± 0.068 ^{aB}	81.298 ± 0.237 ^{acC}	87.318 ± 0.051 ^{aD}	90.888 ± 1.006 ^{aAB}	86.888 ± 2.012 ^{adABCD}	62.653 ± 0.029 ^{cE}	90.193 ± 0.286 ^{aA}

For each column, mean values with the same lower-case letters are not statistically significant during storage ($p > 0.05$). For each row, mean values with the same upper-case letters are not statistically significant among the packaging conditions ($p > 0.05$).

Table S6 Percentage relative humidity levels of packaged breads during the late storage period.

	Packaging Conditions					
**Storage Time**	**C2**	**C3**	**C4**	**T2**	**T3**	**T4**
**Day 6**	88.58 ± 0.043 ^{dA}	83.085 ± 0.12 ^{eB}	87.78 ± 0.047 ^{aC}	75.645 ± 0.168 ^{bcD}	62.778 ± 0.061 ^{aE}	91.865± 0.166 ^{cF}
**Day 9**	89.34 ± 0.057 ^{cA}	84.175 ± 0.021 ^{dB}	87.77 ± 0.00 ^{aC}	75.708 ± 0.063 ^{cD}	63.08 ± 0.022 ^{bE}	93.143 ± 0.031 ^{bF}
**Day 12**	91.1 ± 0.036 ^{bA}	84.84 ± 0.132 ^{cB}	87.77 ± 0.00 ^{aC}	76.09 ± 0.134 ^{bD}	60.343 ± 0.01 ^{cE}	93.343 ± 0.025 ^{aF}
**Day 15**	91.52 ± 0.00 ^{aA}	85.145 ± 0.037 ^{bB}	87.77 ± 0.00 ^{aC}	75.995 ± 0.179 ^{bcD}	59.133 ± 0.062 ^{dE}	93.375 ± 0.042 ^{aF}
**Day 18**	91.52 ± 0.00 ^{aA}	85.808 ± 0.026 ^{aB}	87.77 ± 0.00 ^{aC}	75.235 ± 0.076 ^{aD}	58.633 ± 0.03 ^{eE}	93.385 ± 0.021 ^{aF}

For each column, mean values with the same lower-case letters are not statistically significant during storage ($p > 0.05$). For each row, mean values with the same upper-case letters are not statistically significant among the packaging conditions ($p > 0.05$).

Table S7 Water vapor content in the headspace of packaged breads during the early storage period.

	Packaging Conditions							
**Storage Time**	**C1**	**C2**	**C3**	**C4**	**T1**	**T2**	**T3**	**T4**
**Day 0**	18051.5 ± 1429.91 ^{abA}	21488.75 ± 1769.08 ^{aA}	14540 ± 4929.05 ^{aAB}	21007.25 ± 1917.01 ^{aA}	22455.25 ± 4679.29 ^{aAB}	17807.75 ± 752.81 ^{aA}	15381.25 ± 3690.23 ^{aAB}	12294.5 ± 703.38 ^{bB}
**Day 1**	18471.25 ± 1096.94 ^{abA}	21556.5 ± 1657.66 ^{aA}	14923.75 ± 4439.34 ^{aAB}	16622.75 ± 3260.88 ^{aAB}	19775.75 ± 1513.46 ^{aA}	17955.75 ± 621.63 ^{aA}	17796 ± 1267.72 ^{aA}	13535.25 ± 374.36 ^{abB}
**Day 2**	18826 ± 843.35 ^{bA}	21598 ± 1612.61 ^{aA}	16099.25 ± 4284.22 ^{aAB}	17389 ± 2332.94 ^{aAB}	19444 ± 882.85 ^{aA}	18011.25 ± 645.39 ^{aA}	18481 ± 664.36 ^{aA}	14405.25 ± 1249.43 ^{abB}
**Day 3**	19088.25 ± 672.64 ^{bA}	21682 ± 1508.65 ^{aA}	16238.5 ± 4165.2 ^{aAB}	18166 ± 1601.99 ^{aAB}	19275.25 ± 384.45 ^{aA}	17894.25 ± 802.88 ^{aAB}	18890 ± 642.83 ^{aA}	15127.5 ± 1292.36 ^{aB}
**Day 4**	19388.5 ± 739.25 ^{abAB}	21745 ± 1454.32 ^{aA}	16595.25 ± 3831.97 ^{aAB}	18611 ± 1070.45 ^{aAB}	19339.25 ± 430.25 ^{aAB}	18086.5 ± 614.8 ^{aAB}	19019.5 ± 633.47 ^{aAB}	15610 ± 1468.8 ^{aB}
**Day 5**	19640.5 ± 604.23 ^{aAB}	21775.5 ± 1401.71 ^{aA}	16865.5 ± 3773.27 ^{aAB}	18811 ± 808.74 ^{aAB}	19388 ± 443.9 ^{aAB}	18130.25 ± 530.7 ^{aAB}	19167.75 ± 628.72 ^{aAB}	16309.25 ± 1678.58 ^{aB}

For each column, mean values with the same lower-case letters are not statistically significant during storage ($p > 0.05$). For each row, mean values with the same upper-case letters are not statistically significant among the packaging conditions ($p > 0.05$).

Table S8 Water vapor content in the headspace of packaged breads during the late storage period.

	Packaging Conditions					
**Storage Time**	**C2**	**C3**	**C4**	**T2**	**T3**	**T4**
**Day 6**	21805.75 ± 1369.97 ^{A}	17011.75 ± 3646.67 ^{AB}	19205 ± 1263.74 ^{AB}	18468.25 ± 635.7 ^{AB}	19230.25 ± 629.54 ^{AB}	16578.5 ± 1669.03 ^{B}
**Day 9**	21936.5± 1439.94 ^{A}	15143.75 ± 4578.71 ^{AB}	19822.75 ± 1295.34 ^{AB}	18749.25 ± 1056.52 ^{AB}	19384.25 ± 579.58 ^{AB}	17320 ± 1577.32 ^{B}
**Day 12**	21564.5± 1831.47 ^{A}	16721 ± 3653.09 ^{A}	19962.25 ± 1276.66 ^{A}	18647.25 ± 1013.48 ^{A}	18540.5 ± 1964.63 ^{A}	17836.5 ± 1301.48 ^{A}
**Day 15**	21577.5± 1981.18 ^{A}	15379.25 ± 2589.17 ^{A}	20053 ± 1153.45 ^{A}	18992 ± 927.83 ^{A}	16613.25 ± 3109.5 ^{A}	18845.5 ± 1345.25 ^{A}
**Day 18**	21641± 1906.72 ^{A}	16146.25 ± 2459.59 ^{A}	20176.75 ± 1122.79 ^{A}	18708.75 ± 738.68 ^{A}	16492.75 ± 2663.95 ^{A}	19617.5 ± 1268.01 ^{A}

For each row, mean values with the same upper-case letters are not statistically significant among the packaging conditions ($p > 0.05$).

Table S9 Dew point temperature of packaged breads during the early storage period.

	Packaging Conditions							
**Storage Time**	**C1**	**C2**	**C3**	**C4**	**T1**	**T2**	**T3**	**T4**
**Day 0**	18.943 ± 3.693^{ACD}	15.853 ± 1.418^{AD}	6.985 ± 0.732^{B}	19.255 ± 0.064^{A}	22.995 ± 0.248^{C}	12.27 ± 1.491^{D}	11.893 ± 3.218^{ABD}	12.958 ± 2.273^{ABD}
**Day 1**	15.443 ± 1.767^{AC}	17.215 ± 1.247^{AC}	6.998 ± 0.217^{B}	17.415 ± 2.468^{AC}	18.94 ± 1.353^{C}	14.99 ± 0.396^{AC}	14.685 ± 0.991^{A}	13.595 ± 1.867^{A}
**Day 2**	15.835 ± 1.054^{A}	17.645 ± 1.047^{A}	7.163 ± 0.03^{B}	16.433 ± 2.259^{A}	16.9 ± 0.691^{A}	15.643 ± 0.067^{A}	15.498 ± 0.721^{A}	14.048 ± 1.81^{A}
**Day 3**	16.345 ± 0.808^{AC}	18.025 ± 0.586^{A}	7.228 ± 0.049^{B}	14.578 ± 1.627^{AC}	16.445 ± 0.186^{A}	15.75 ± 0.048^{C}	15.948 ± 0.778^{AC}	14.49 ± 1.753^{AC}
**Day 4**	16.878 ± 0.321^{AC}	18.17 ± 0.556^{A}	7.365 ± 0.142^{B}	14.808 ± 1.267^{ACD}	16.543 ± 0.4^{CD}	15.828 ± 0.075^{D}	16.225 ± 0.876^{ACD}	15.158 ± 1.097^{ACD}
**Day 5**	17.008 ± 0.345^{AC}	18.223 ± 0.479^{A}	7.53 ± 0.178^{B}	15.378 ± 0.877^{CD}	16.848 ± 1.012^{ACD}	15.863 ± 0.083^{D}	16.433 ± 1.023^{ACD}	15.445 ± 0.915^{CD}

For each row, mean values with the same upper-case letters are not statistically significant among the packaging conditions ($p > 0.05$).

Table S10 Dew point temperature of packaged breads during the late storage period.

	Packaging Conditions					
**Storage Time**	**C2**	**C3**	**C4**	**T2**	**T3**	**T4**
**Day 6**	18.265 ± 0.401^{bA}	7.725 ± 0.236^{dB}	15.713 ± 0.954^{aC}	15.853 ± 0.109^{aC}	16.548 ± 1.096^{abAC}	15.698 ± 0.752^{aC}
**Day 9**	18.485 ± 0.473^{abA}	8.283 ± 0.311^{cB}	16.263 ± 0.812^{aC}	15.685 ± 0.03^{aC}	16.845 ± 1.29^{bAC}	16.138 ± 0.801^{aC}
**Day 12**	18.71 ± 0.554^{abA}	9.188 ± 0.319^{bcB}	16.628 ± 0.251^{aC}	15.695 ± 0.134^{aD}	16.728 ± 1.3^{aACD}	16.643 ± 0.937^{aACD}
**Day 15**	18.843 ± 0.526^{aA}	9.748 ± 0.212^{abB}	16.833 ± 0.102^{aC}	16.253 ± 0.549^{aC}	16.278 ± 1.124^{abAC}	17.343 ± 1.022^{aAC}
**Day 18**	19.078 ± 0.118^{abA}	10.848 ± 0.832^{aB}	17.105 ± 0.113^{aC}	16.453 ± 0.617^{aC}	16.205 ± 1.2^{abAC}	17.678 ± 1.007^{aAC}

For each column, mean values with the same lower-case letters are not statistically significant during storage ($p > 0.05$). For each row, mean values with the same upper-case letters are not statistically significant among the packaging conditions ($p > 0.05$).
